# Adaptation of Endoluminal Vacuum Therapy via Extra-Luminal Access in the Treatment of a High Pharyngeal Fistula: Technical Feasibility and Outcome

**DOI:** 10.3390/life15111660

**Published:** 2025-10-23

**Authors:** Bogdan-Mihnea Ciuntu, Daniel Vasile Timofte, Andreea Ludușanu, Mihaela Corlade-Andrei, Roxana Elena Ciuntu, Irina Mihaela Abdulan, Alexandra-Simona Zamfir, Adelina Tanevski, Mădălina Maxim, Gheorghe Balan, Anca Bordianu, Bogdan Cobzeanu

**Affiliations:** 1 Department of General Surgery, Faculty of Medicine, “Grigore T. Popa” University of Medicine and Pharmacy, 16 Universitatii Street, 700115 Iasi, Romania; bogdan-mihnea.ciuntu@umfiasi.ro (B.-M.C.); dantimofte@yahoo.com (D.V.T.); andreealudusanu1106@yahoo.com (A.L.);; 2 General Surgery Clinic, “St. Spiridon” County Emergency Clinical Hospital, 1 Independence Boulevard, 700111 Iasi, Romania; 3 Department of Emergency Medicine, Surgery II, “Grigore T. Popa” University of Medicine and Pharmacy, 700115 Iasi, Romania; mihaela.corlade2@umfiasi.ro; 4 Department of Ophtalmology, “Grigore T. Popa” University of Medicine and Pharmacy, 16 Universitatii Street, 700115 Iasi, Romania; roxana-elena.ciuntu@umfiasi.ro; 5 Department of Medical Specialties I, “Grigore T. Popa” University of Medicine and Pharmacy, 700115 Iasi, Romania; 6 Department of Medical Sciences III, Pulmonology, Faculty of Medicine, “Grigore T. Popa” University of Medicine and Pharmacy, 700115 Iasi, Romania; 7 Department of Gastroenterology, “Grigore T. Popa” University of Medicine and Pharmacy, 700115 Iasi, Romania; gheorghe-g-balan@umfiasi.ro; 8 Department of Vascular Surgery, Faculty of Medicine, “Grigore T. Popa” University of Medicine and Pharmacy, 16 Universitatii Street, 700115 Iasi, Romania; bordianu.anca@d.umfiasi.ro; 9 ENT Clinic Department, “Grigore T. Popa” University of Medicine and Pharmacy, 700115 Iasi, Romania; bogdan788@yahoo.com

**Keywords:** piriform sinus, perforation, mediastinitis, dental prosthesis ingestion, EVAC, deep neck infection, foreign body, sepsis, tracheostomy

## Abstract

Background: Perforation of the piriform sinus is a rare but severe clinical event that can lead to cervico-thoracic mediastinitis, a life-threatening condition requiring urgent multidisciplinary intervention. Among its etiologies, accidental ingestion of foreign bodies, including dental prostheses, is uncommon but poses significant risks due to the anatomical vulnerability of the hypopharyngeal structures. Methods: We report a rare case of right piriform sinus perforation secondary to the ingestion of a dental prosthesis, complicated by cervico-mediastinitis, sepsis, tracheostomy, and sacral pressure ulcer. The clinical course required emergency surgical intervention and intensive supportive care. Results: A novel aspect of this case was the use of the Endoscopic Vacuum-Assisted Closure (EVAC) irrigation system as an adjunctive technique in the management of deep cervical drainage. Rather than approaching the fistula from within the lumen, the team created a controlled external drainage system, adaptation of the vacuum-assisted closure therapy directly over the fistulous tract. Conclusions: This case highlights the importance of early diagnosis, high clinical suspicion and coordinated management in the treatment of piriform sinus perforations. It also illustrates the potential applicability of modern technologies such as negative pressure irrigation in the complex management of deep neck infections and mediastinitis.

## 1. Introduction

Accidental perforation of the piriform sinus, a recess located in the hypopharynx, can result in acute cervico-thoracic mediastinitis, a rare but potentially life-threatening condition, often triggered by the ingestion of a foreign body [1]. This clinical scenario demands a high index of suspicion, as well as immediate and coordinated management by a multidisciplinary team comprising otolaryngology, pulmonology, thoracic surgery and infectious disease specialists, in order to ensure favorable patient outcomes [2]. Due to its anatomical location and the biomechanics of swallowing, the piriform sinus is particularly susceptible to perforation by sharp or irregularly shaped foreign bodies [3]. Although mediastinitis may arise from various causes—such as esophageal rupture or postoperative complications—perforation of the piriform sinus secondary to foreign body ingestion remains an uncommon but equally serious etiology [3,4]. The inflammatory cascade triggered by such perforation can progress rapidly, leading to cervico-mediastinitis, characterized by infection and inflammation that extend from the neck into the mediastinum.

Early recognition and prompt intervention are critical to avert life-threatening complications such as sepsis, airway compromise and mediastinal abscess formation [5]. The piriform sinus, an anatomical structure situated on either side of the laryngeal inlet within the lower pharynx, plays a vital role in channeling food into the esophagus while protecting the airway [5,6].

Although piriform sinus perforations are rare, they may occur in traumatic, iatrogenic, or tumoral contexts, or following foreign body ingestion, and are frequently associated with serious complications such as mediastinitis, aerodigestive fistulas, and sepsis [6]. Among iatrogenic causes, dental or surgical interventions in the oropharyngeal region may—under exceptional circumstances—lead to sinus perforation, particularly in the presence of risk factors such as unstable dental prostheses, aggressive manipulation or poor control over the upper respiratory-digestive junction [7].

Accidental ingestion of a dental prosthesis represents a medical emergency with unpredictable outcomes depending on the object’s morphology and location. In severe cases, this may lead to pharyngeal perforation, cervico-thoracic mediastinitis, and even respiratory failure, necessitating emergency surgery and complex multidisciplinary care [8].

This article presents a rare clinical case of right piriform sinus perforation following the ingestion of a dental prosthesis, complicated by sepsis, tracheostomy and sacral pressure ulcer. Notably, the EVAC system was used as an adjunctive technique for deep cervical drainage, representing an innovative approach in the management of this severe complication. A modified vacuum-assisted closure (VAC) system was employed, and adapted for external application over a high pharyngoesophageal fistula, where standard endoluminal approaches such as stenting or EVAC aremetallic technically unfeasible. This extra-luminal approach allowed for the creation of a controlled drainage tract with progressive closure from the outside, representing an innovative method for managing complex upper digestive fistulas. The case underscores the critical importance of early diagnosis, interdisciplinary collaboration, and the adaptation of modern technologies in addressing extreme clinical scenarios.

## 2. Case Report

A 45-year-old male patient presented to the Emergency Department of “Sf. Spiridon” County Emergency Hospital in Iași with complaints of severe dysphagia, odynophagia, and high-grade fever. The onset of symptoms was reported following the accidental ingestion and subsequent extraction of a dental prosthesis (Kemenny with metallic base).

On admission, the patient was in a seriously deteriorated general condition, with a body temperature of 39 °C, blood pressure of 114/75 mmHg, and a heart rate of 77 bpm. Respiratory evaluation revealed a respiratory rate of 17 breaths per minute and oxygen saturation of 98% on room air, indicating early signs of respiratory compromise, though not yet decompensated.

Laboratory investigations obtained at the time of admission revealed significant markers of systemic inflammation (Table 1).

These findings are consistent with systemic inflammatory response syndrome (SIRS) progressing towards sepsis, as defined by leukocytosis, elevated CRP, metabolic disturbances and signs of organ dysfunction.

Flexible naso-pharyngolaryngoscopy revealed a deviated nasal septum to the right with partial obstruction. The posterior pharyngeal wall, including the nasopharynx, oropharynx and hypopharynx, showed no ulcerations or significant congestion. However, notable salivary stasis with a fetid odor was observed in the piriform sinuses, raising suspicion of pooling due to local inflammation or impaired drainage. The glottis was free, and the vocal cords were mobile, suggesting no immediate airway obstruction.

Contrast-enhanced cervico-thoracic computed tomography revealed a well-defined hydro-aerial collection with an iodophilic wall measuring up to 4 mm in thickness and overall dimensions of approximately 64 × 35 × 170 mm (transverse × anteroposterior × craniocaudal). The collection was located posterior to the oropharynx, extending inferiorly into the upper and middle mediastinum. Anatomical boundaries of the collection were limited by a horizontal plane passing through the C2–C3 intervertebral space, extending to the level of the T6 vertebral body, in close proximity to the esophagus, which appeared collapsed, likely due to a mass effect; esophageal wall continuity could not be reliably assessed and in contact with the vertebral bodies, without evidence of bone erosion or disruption.

The collection was observed to be in contact with critical vascular structures, such as the right subclavian artery, the vertebral arteries and the common carotid arteries bilaterally, as well as the aortic arch and descending thoracic aorta. Additionally, contact was noted with the postero-superior aspect of both thyroid lobes.

At the level of the bilateral piriform sinuses, two symmetrical air pockets (~8 mm) were detected at approximately 9 mm from the hypopharyngeal lumen. Notably, the right piriform sinus showed direct communication with the aforementioned mediastinal collection. Furthermore, a 6 mm gas bubble was identified anterosuperior to the right piriform sinus, adjacent to the posterior surface of the thyrohyoid muscle.

Additional findings included the presence of antero-superior pneumomediastinum and bilateral supra-pleural emphysema, consistent with air dissection along fascial planes secondary to pharyngeal perforation.

Based on clinical presentation and imaging findings, we established the diagnosis of iatrogenic perforation of the right piriform sinus complicated by cervico-thoracic mediastinitis.

The patient was admitted to the surgical department, where he underwent a series of emergency surgical procedures due to the extent of the infection and the risk of complications. The surgical approach included a right lateral cervicotomy for drainage of the cervico-mediastinal collection, followed by a median laparotomy with transhiatal mediastinal drainage, which was supplemented by additional cervical drainage. To ensure adequate nutritional support, a feeding jejunostomy was placed, and a subhepatic drainage tube was inserted for abdominal fluid management.

Postoperatively, the patient required intensive care unit admission with mechanical ventilation, targeted antimicrobial therapy, enteral nutrition, and continuous radiological monitoring using serial CT scans.

Microbiological analysis of the mediastinal collection identified Streptococcus anginosus and Acinetobacter baumannii. Based on the antibiogram, the patient received a tailored antibiotic regimen comprising intravenous administration of carbapenem 1 g/day, metronidazole 2 g/day vancomycin 2 g/day and orally fluconazole 100 mg/day.

Despite the severe clinical context, hemocultures remained negative, and antifungal therapy was continued empirically. Nutritional support was ensured via enteral feeding through jejunostomy, and the evolution was monitored closely by repeated computed tomography imaging to assess resolution of the mediastinal and cervical collections.

Despite the initial surgical management and intensive care, the patient’s clinical evolution remained unfavorable, with persistent sepsis and elevated inflammatory markers. Follow-up contrast-enhanced computed tomography imaging revealed a right supradiaphragmatic fluid collection with a thickness of 86 mm, as well as a right subdiaphragmatic, oval-shaped fluid formation with homogeneous content, measuring 160 × 80 × 151 mm (transverse × anteroposterior × craniocaudal).

**Figure 1:**
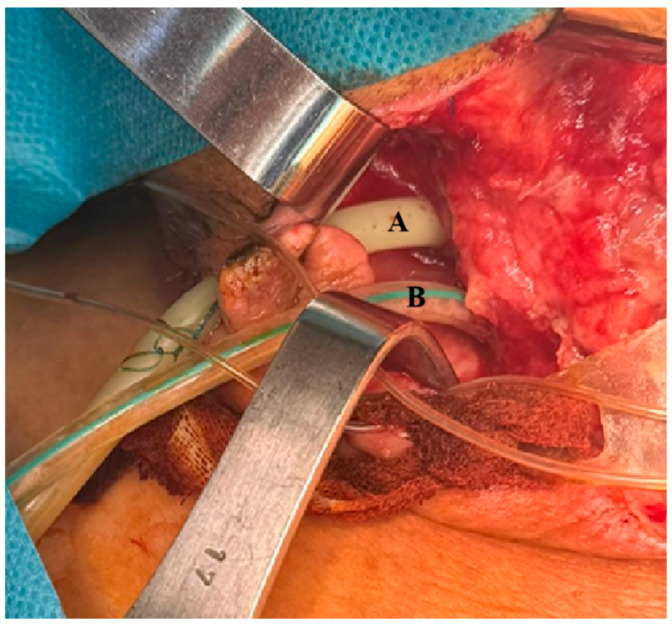
(**A**) Deep cervical drainage tube and (**B**)—externally adapted VAC double-lumen nasogastric tube.

At the thoracic level, additional findings included a focal ulceration (~2 mm) of the posterolateral wall at the cervico-thoracic junction, located adjacent to the balloon of the endotracheal tube. These features were suggestive of tracheal injury secondary to prolonged mechanical ventilation. As a result, the patient underwent a relaparotomy with extensive peritoneal and mediastinal lavage, along with the replacement of existing drainage tubes. Given the need for prolonged mechanical ventilation and the presence of tracheal wall injury, a tracheostomy was performed. In additions a customized vacuum-assisted closure (VAC) system was implemented, specifically adapted for external application to address the high pharyngoesophageal fistula, in a clinical context where conventional endoluminal approaches—such as stent placement or EVAC—, are technically impractical (Figure 1).

This complex surgical and critical care approach aimed to control the source of sepsis, support respiratory function and enhance local wound management in the context of deep neck and mediastinal infection.

For the implementation of the externally adapted Vacuum-Assisted Closure (VAC) system technique, a customized system was assembled comprising a double-lumen nasogastric tube, a size S polyurethane foam sponge (vacuum kit), standard surgical instruments, and a vacuum aspiration pump. The sponge was carefully cut and shaped to conform to the exact dimensions of the infected cavity. The nasogastric tube was prepared accordingly, ensuring that the aspiration ports aligned precisely with the proximal and distal ends of the sponge. A longitudinal incision along the central axis of the sponge was performed to accommodate the probe, allowing its insertion and stabilization within the foam structure.

This specialized configuration enables simultaneous irrigation, the local administration of therapeutic agents and controlled negative pressure drainage while also facilitating the monitoring of cavity integrity and tightness (Table 2).

The procedure required a multidisciplinary team, including an endoscopist, surgeon, and anesthesiologist, and was performed under general anesthesia with orotracheal intubation, in a sterile operating room environment. After standard preoperative preparations, an upper digestive endoscopy was conducted to localize the defect, irrigate the cavity, and assess its extent and walls.

The nasogastric tube was initially introduced through the lateral cervicectomy, and the endoscope was then used to guide the sponge-loaded probe back toward the identified defect. The assembly was advanced carefully into the cavity using the piggyback technique.

Proper intraluminal and intracavitary positioning was verified by gentle endoscopic retraction, followed by activation of the vacuum system, with negative pressure settings ranging between 150 mmHg and 170 mmHg. Once correct placement was confirmed visually, the endoscope was withdrawn, and the nasogastric tube was fixed externally to prevent displacement.

**Figure 2:**
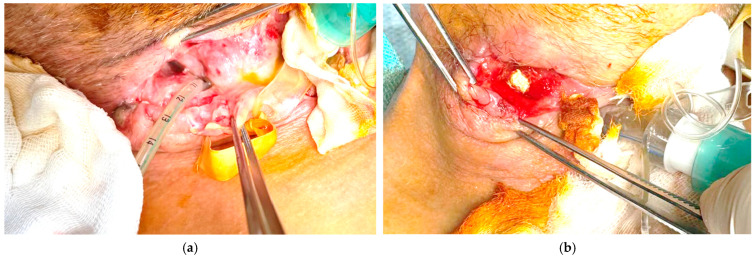
Local evolution of the cervical cavity. (**a**) Local evolution of the cervical cavity at the second E-VAC change. (**b**) Local evolution of the cervical cavity in the middle of the E-VAC treatment period.

The clinical evolution was gradually favorable, necessitating the periodic replacement of the external VAC system every 5 days, under continuous computed tomography imaging and paraclinical monitoring to assess cavity reduction and prevent complications (Figure 2).

**Figure 3:**
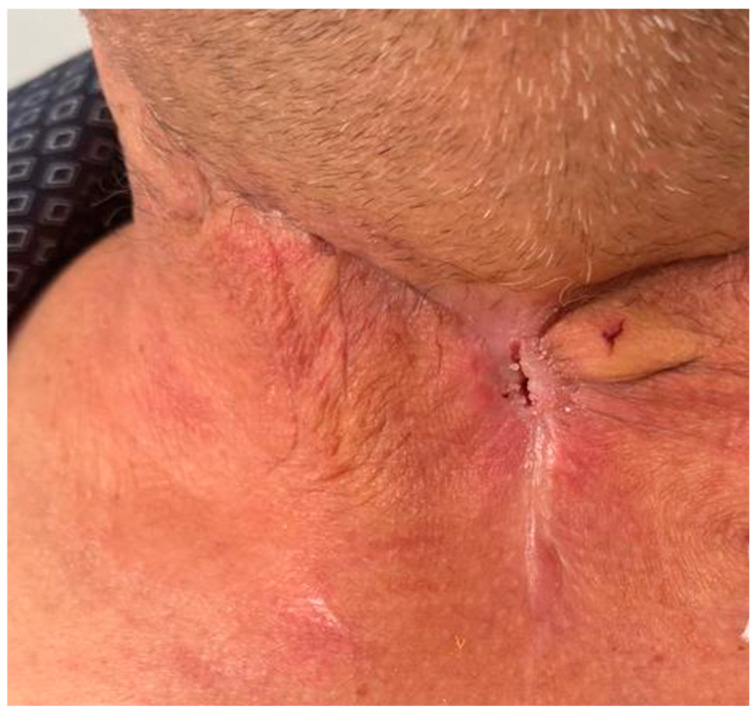
Postoperative outcome of the cervical scar (after one month after discharge).

The patient was discharged after a prolonged hospitalization of over two months, in improved general condition, with instructions for periodic follow-up (Figure 3).

## 3. Discussion

The promising outcomes achieved through endoluminal negative aspiration in treating high digestive fistulas, coupled with enhancements to the aspiration system that allow for lavage at the site of the defect and ensure its impermeability, have prompted the adaptation of this technique for fistulas located in the upper esophagus. This method, which can be performed using moderate sedation endoscopy without the need for intubation, necessitates fewer changes and is better accepted by patients [9]. The placement of esophageal stents offers a non-invasive strategy for the management of gastro-mediastinal or gastro-pleural fistulas that arise post-esophagogastrectomy [10]. However, challenges such as stent displacement, ongoing leakage, and the potential development of esophagotracheal fistulas have been reported [11]. Endoscopic vacuum therapy has emerged as a minimally invasive alternative for addressing intestinal defects, involving the strategic placement of sponge drainages at varying levels within the gastrointestinal tract [12,13]. This approach facilitates continuous drainage and promotes defect closure through the application of consistent negative pressure, typically around 125 mmHg [13].

Given the technical challenges associated with an endoscopic approach to these fistulas, an alternative strategy is required for this segment of the digestive tract. The preparation of the kit is like that used in endoscopic negative pressure wound therapy; however, it is positioned at the esophageal defect outside the lumen, under direct visualization, via a latero-cervical approach. Upon initial placement, the sponge, which is prepared according to the dimensions of the defect, makes direct contact with it and is then gradually retracted toward the wound’s edges. The primary goal of the initial placement is to aspirate the entire wound surface and enhance local vascularization. This facilitates the progressive closure of the defect in contact with the probe’s distal end during subsequent retraction stages. Early investigations have indicated the viability and safety of endoscopic vacuum therapy for addressing esophageal defects, demonstrating its capacity to facilitate defect closure through sustained drainage and tissue approximation [14,15].

The healing stages were similar to those observed in endoluminal treated defects. This approach, applicable to lesions located in the upper third of the esophagus, which until now could not be treated using the endoscopic approach, demonstrates its usefulness in the minimally invasive management of these lesions, offering favorable results. One study reported the successful management of post-esophagectomy anastomotic fistulas using endoscopic trans-fistula negative pressure drainage, highlighting its convenience and reliability in promoting fistula closure [15]. Further investigation is warranted to refine techniques and optimize outcomes in diverse clinical scenarios. The application of a wound vac sponge sutured to a nasogastric tube, positioned at the perforation site under fluoroscopic guidance and connected to continuous low suction, has shown promise in resolving leaks and promoting healing [16]. The use of modified drainage systems, such as a nasogastric tube with gauze coated in perforated sterile plastic, can also provide a low-cost alternative for applying negative pressure [17]. This method necessitates a multidisciplinary approach, involving cardiothoracic surgery and gastroenterology, alongside nutritional support via percutaneous gastric or jejunal tubes [18].

In addition to facilitating local treatment and infection control, the benefit of second lumen lavage lies in its extended duration, allowing for the kit to remain in situ for up to 8 to 9 days, thereby decreasing the necessity for repeated general anesthesia. While some researchers advocate for early surgical closure of open abdominal surgical site infections, incorporating wound irrigation with negative pressure wound therapy, alternative treatment modalities, such as primary anastomosis, are also viable options [19,20]. The EVAC system offers advantages, including the elimination of nasogastric tubes for decompression or naso-enteric tubes for nutritional support, enabling the continuation of oral feeding and reducing the frequency of dressing changes [21].

Vacuum-assisted closure systems have demonstrated efficacy in the management of enterocutaneous fistulae, supporting wound healing and managing fistula drainage [21,22]. However, the considerable expenses linked to vacuum-assisted closure systems and related nursing interventions should be considered [22].

A significant aspect of the multidisciplinary therapeutic strategy involved the utilization of the EVAC technique—a negative pressure endoluminal irrigation system modified for the purpose of draining and cleansing the deep cervical region [22,23]. The implementation of EVAC facilitated enhanced infection control, secretion removal, and the acceleration of tissue repair [23]. This method offers an efficient and cost-effective approach to applying negative pressure wound therapy in hand surgery facilitating drainage while simultaneously monitoring local vascularization [24].

An important innovation in this case lies in the adaptation of vacuum-assisted therapy for the treatment of high digestive tract fistulas—an anatomical region where standard therapeutic approaches, such as endoluminal stenting or conventional EVAC, are technically challenging or outright infeasible. The anatomical constraints of the upper pharyngo-esophageal segment often preclude the effective placement of stents due to poor anchoring and high mobility, and similarly hinder the application of traditional intraluminal vacuum systems.

In this context, the clinical team employed a novel approach: the external adaptation of vacuum-assisted closure therapy directly over the fistulous tract. Rather than approaching the fistula from within the lumen, the team created a controlled external drainage system, allowing for continuous negative pressure application from outside the digestive tract. This strategy enabled progressive closure of the fistulous pathway, while ensuring effective drainage, infection control and tissue granulation.

## 4. Conclusions

The effective management of surgical complications requires not only targeted treatment of the complication itself, but also a comprehensive assessment of the patient’s overall physiological status. Optimal outcomes are best achieved through a collaborative, multidisciplinary approach, drawing on the combined expertise of surgeons, gastroenterologists, radiologists, and dietitians. In recent years, EVAC has emerged as a promising adjunct in the treatment of anastomotic leaks and other gastrointestinal fistulas. However, surgical re-intervention remains indispensable in selected cases where endoscopic therapy is insufficient.

Close clinical surveillance and structured follow-up protocols are essential components of care, enabling the early detection of treatment failure and facilitating timely salvage interventions. Such proactive management strategies have been associated with improved outcomes, especially in cases of fistulas that demonstrate delayed or incomplete healing.

This case thus demonstrates a paradigm shift in the management of complex pharyngoesophageal fistulas—transforming a typically inoperable and anatomically inaccessible condition into one that is actively and safely managed via external vacuum application, tailored to the specific topography of the lesion.

## Figures and Tables

**Table 1:** Laboratory parameters.

Laboratory Investigations	Values	Normal Values
Leukocytes	19.64 × 10^3^/μL	4.0–10.0 × 10^3^/μL
C-reactive protein	36 mg/dL	0–0.5 mg/dL
Neutrophils	18.62 × 10^3^/μL	2.0–8.0 × 10^3^/μL
Lymphocytes	0.41 × 10^3^/μL	1.0–4.0 × 10^3^/μL
Hemoglobin	11.2 g/dL	13.0–17.3 g/dL
Hematocrit	31.3%	39.0–51.0%
Urea	50 mg/dL	13–43 mg/dL
Potassium	3.2 mmol/L	3.5–5.1 mmol/L
Alkaline reserve	20.5 mmol/L	22–29 U/L

**Table 2:** Key settings and adjunctive care in Vacuum Therapy.

Parameter	Value/Range
Vacuum pressure applied	150 mmHg to −170 mmHg
Sponge replacement interval	Every 4–5 days
Endoscopic confirmation of placement	Mandatory at each replacement
Nutritional support	Enteral (via jejunostomy)
Additional therapy	Broad-spectrum antibiotics, Proton Pump Inhibitors

## Data Availability

The original contributions presented in this study are included in the article. Further inquiries can be directed to the corresponding author.
